# Nutrient consumption-dependent association of a glucagon-like peptide-1 receptor gene polymorphism with insulin secretion

**DOI:** 10.1038/s41598-020-71853-7

**Published:** 2020-10-02

**Authors:** Yuki Nishiya, Makoto Daimon, Satoru Mizushiri, Hiroshi Murakami, Jutaro Tanabe, Yuki Matsuhashi, Miyuki Yanagimachi, Itoyo Tokuda, Kaori Sawada, Kazushige Ihara

**Affiliations:** 1 Department of Endocrinology and Metabolism, Hirosaki University Graduate School of Medicine, Hirosaki, Aomori Japan; 2 Department of Oral Healthcare Science, Hirosaki University Graduate School of Medicine, Hirosaki, Aomori Japan; 3 Department of Social Medicine, Hirosaki University Graduate School of Medicine, Hirosaki, Aomori Japan

**Keywords:** Endocrinology, Health care, Molecular medicine, Risk factors

## Abstract

Since type 2 diabetes (DM) is a life-style related disease, life-style should be considered when association between genetic factors and DM are examined. However, most studies did not examine genetic associations in consideration with lifestyle. Glucagon-like peptide-1 (GLP-1) receptor (GLP1R) mediates the insulinotropic action of GLP-1 in β-cells. We here examined the association while taking into consideration of interactions between the gene polymorphism and various nutrient factors. Participants from the population-based Iwaki study of Japanese subjects held in 2014–2017 with information on nutritional intake evaluated by self-administered dietary history questionnaire, and GLP1R genotype (rs3765467: A/G), were included (n = 1,560). Although not significant, insulin secretion indices assessed by homeostasis model assessment of β-cell function (HOMA-β) in subjects with the GG genotype tended to be lower than in those with the AA+AG genotypes in most groups stratified into tertiles based on daily nutrient consumptions (high, middle, and low). Stratification also showed that the GG genotype was a significant risk for decreased insulin secretion (HOMA-β ≤ 30) even after adjustment for multiple factors (age, body mass index, alcohol consumption), but only in the highest tertiles of energy, protein and carbohydrate consumption in men [odds ratios (95% confidence interval) 3.95 (1.03–15.1), 15.83 (1.58–158.9), and 4.23 (1.10–11.2), respectively]. A polymorphism of the GLP1R gene was associated with decreased insulin secretion in a nutrient consumption-dependent manner in Japanese men, indicating an interaction between GLP1R and nutritional factors in the pathophysiology of DM.

## Introduction

Type 2 diabetes (DM), a heterogeneous disorder of glucose metabolism characterized by both insulin resistance and pancreatic β-cell dysfunction, is considered multifactorial, as many genetic and environmental factors are involved together in its pathophysiology^1,2^. Therefore, thorough understanding such factors is important as it may promote development of individualized or precision medicine. However, although many genes have been identified as DM susceptibility genes across several studies (including genome wide association studies (GWASs)^3–7^, the most powerful and stringent methods for identifying the genetic basis of common diseases), most of the relevant information has not been used to inform decision-making in the general clinical setting^8^. One reason why such data are insufficient for use is the lack of corresponding information on factors that interact with the reported genetic factors, since the pattern of inheritance of DM suggests not only polygenic inheritance, but also a complex genetic interaction with environmental factors^1,2,9,10^.

Glucagon-like peptide-1 (GLP-1), secreted from enteroendocrine L-cells of the intestine, enhances insulin secretion in a glucose-dependent manner via its cognate receptor, GLP1R. Therefore, various GLP-1 based therapies (e.g. dipeptidyl peptidase-4 inhibitors and GLP1R agonists) have been applied to treat DM with adequate effects^11,12^. Association between GLP1R gene polymorphisms and DM has also been reported in various case–control studies, including GWASs^13–18^. Therefore, the effects of GLP-1 based therapies may vary depending on genotypes of the GLP1R gene. Evaluating the relationship between the effects of such therapies and GLP1R genotypes may be useful for identifying subjects suitable for GLP-1 based therapy. Indeed, the functional relevance of several GLP1R gene polymorphisms regarding the effects of GLP-1 administration have been reported^19,20^. Among them, the polymorphism, rs3765467 (A/G: p.Arg131Gln), was shown to be functional with the A allele associated with a > 100% increase in GLP-1 induced insulin secretion^19^. Taken together, these observations indicate that GLP1R is a DM susceptibility gene.

Therefore, interactions between the GLP1R gene and environmental factors are also highly important. GLP-1 secretes in response to administration of glucose and various nutrients including fat and amino acids^21,22^. Therefore, the amounts of such various nutrients consumed may affect the association between GLP1R genotype and DM. To date, no studies examined such interaction.

To analyze this matter in details, we here examined the interaction between nutrients consumed and the association of the GLP1R gene with impaired glucose metabolism or decreased insulin secretion, in a population-based sample of Japanese subjects. Our findings may be useful to find subjects who are susceptible to decreased insulin secretion in nutrient consumption-dependent manner.

## Methods

### Study population

Subjects were recruited from the Iwaki study, a health promotion study of Japanese people over 20 years old aimed at preventing lifestyle-related diseases and prolonging lifespans. The study is conducted annually in the Iwaki area of the city of Hirosaki in Aomori Prefecture in northern Japan^23,24^. Among 1,817 participants in the Iwaki study held in 2014–2017, 1,676 individuals were considered eligible for the present study, as they have complete data on genotype of the GLP1R polymorphism (db SNP ID: rs3765467: A/G) (p.Alg131Gln) and nutrient consumption. The following individuals were excluded: 64 on medication for DM (diabetic individuals on diet treatment are included), and 52 with fasting blood glucose (FBG) levels below 63 mg/dl or over 140 mg/dl (to precisely evaluate homeostasis model assessment (HOMA) indices). After these exclusions, 1,560 individuals (587 men and 973 women) aged 53.3 ± 16.1 years were included in the study.

This study was approved by the Ethics Committee of the Hirosaki University School of Medicine (No. 2014-014 and 2014-015), and was conducted according to guidelines of the Declaration of Helsinki. Written informed consent was obtained from all participants.

### Characteristics measured and genotyping

Characteristics were measured as previously reported^23–25^. Namely, peripheral vein blood samples were collected in the morning from participants under fasting conditions in the supine position for 5 min after 10 min rest in a sitting position. The following clinical characteristics were measured: height, body weight, body mass index (BMI), percent body fat (fat), fasting blood glucose, fasting serum insulin levels, glycated hemoglobin (HbA1c), systolic and diastolic blood pressures, serum levels of total cholesterol, triglyceride (TG), high-density lipoprotein-cholesterol, uric acid, urea nitrogen, and creatinine. Fat was measured by the bioelectricity impedance method with a Tanita MC-190 body composition analyzer (Tanita Corp., Tokyo, Japan). HbA1c (%) is expressed as the National Glycohemoglobin Standardization Program value. All laboratory testings were performed in a commercial laboratory (LSI Medience Co., Tokyo, Japan) according to vendor protocols. Insulin secretion was evaluated with homeostasis model assessment of β-cell function (HOMA-β), based on fasting blood glucose and insulin levels^26^. Insulin resistance was also assessed based by homeostasis model assessment (HOMA-R)^26^. Daily nutritional intake was estimated using the brief self-administered diet history questionnaire (BDHQ), which is a well-annotated structured self-administered questionnaire invented for Japanese adults to estimate the daily intakes of energy, and selected nutrients by assessing dietary habits during the preceding month^27–30^. In this study, we used values of macronutrients only. Fat consumption was composed of animal and vegetable fat consumptions. Alcohol was not considered as a component of carbohydrate, but fibers were. For energy evaluation, amount of alcohol multiplied by 7.1. DM was defined according to 2010 criteria of the Japan Diabetes Society, i.e. FBG ≥ 126 mg/dl (n = 16)^31^. In subjects where FBG levels were not measured, diabetes was defined as HbA1c ≥ 6.5%. No subjects in our study were known to have type 1 diabetes. Hypertension was defined as blood pressure ≥ 140/90 mmHg or undergoing treatment for hypertension (n = 574). Hyperlipidemia was defined as total cholesterol ≥ 220 mg/dl, TG ≥ 150 mg/dl, or undergoing treatment for hyperlipidemia. (n = 690). Alcohol consumption (current or nondrinker) and smoking habits (never, past or current) were determined from questionnaires.

Genomic DNA was extracted from peripheral whole blood using QIAamp 96 DNA Blood Kit (QIAGEN, Hilden, Germany) and genotypes of the single nucleotide polymorphism (SNP) of the GLP1R gene, rs3765467, were determined by Toshiba corporation using Japonica Array^32^. The SNP was chosen as representative of the GLP1R gene for its association with DM as was reported in previous GWASs, and was shown by structural analysis of crystallized GLP1R to reside in an active site necessary to maintain GLP1R protein structure or to be a functional SNP^19,20^.

### Statistical analysis

Data are presented as means ± SD. The statistical significance of the differences in values between two groups (parametric) and case–control associations between groups (nonparametric) were assessed using analysis of variance and the χ^2^ test, respectively. The independent association of the polymorphism from age, body mass index (BMI), and alcohol consumption was examined by analysis of covariance (ANCOVA) and multiple logistic regression analysis for parametric and non-parametric data, respectively, in each sex. Risk for decreased insulin secretion was evaluated by multiple logistic regression analysis with adjustment for factors described above. For statistical analyses, HOMA indices were log-transformed (log10) to approximate a normal distribution. p < 0.05 was considered statistically significant. All analyses were performed using JMP version 14.0 (SAS Institute Japan Ltd., Tokyo, Japan).

## Results

### Clinical characteristics of study subjects

The clinical characteristics of subjects based on the genotype are shown in Table 1. The proportion of subjects who were currently alcohol drinkers was higher in subjects with the GG genotype than with the genotypes AA+AG (46.5% vs. 40.9%, p = 0.046). However, no other characteristics measured, including HOMA indices, were different between the AA+AG and GG genotypes. Differences in nutrients consumed between the genotypes were then evaluated separately in men and women, since the amounts of nutrients consumed were substantially different between sexes (i.e. protein and fat consumed (g/kgBW/day) were 1.14 ± 0.46 and 1.27 ± 0.55 (p < 0.0001), and 0.83 ± 0.37 and 0.97 ± 0.41 (p < 0.01), respectively, for men and women, respectively). As shown, no differences were observed in nutrients consumed between the genotypes, either in men or women (Table 2). These observations indicate that subjects with the GG genotype may prefer to consume alcohol, but not other macronutrients or total energy.

**Table 1:** Characteristics of study subjects based on GLP1R1 genotype.

Characteristics	AA+AG	GG	p
Number (gender: M/F)	440 (164/276)	1,120 (423/697)	0.86
Age (years)	53.6 ± 16.1	53.2 ± 16.2	0.66
Height (cm)	160.3 ± 9.2	160.7 ± 9.5	0.48
Body weight (kg)	59.4 ± 12.0	8.5	0.75
Body mass index (kg/m^2^)	23.0 ± 3.4	22.8 ± 3.6	0.34
Fat (%)	26.8 ± 8.2	25.9 ± 8.3	0.07
Fasting plasma glucose (mg/dl)	89.3 ± 12.3	89.2 ± 11.7	0.78
HbA1c (%)	5.64 ± 0.39	5.65 ± 0.37	0.75
Fasting serum insulin: IRI (μU/ml)	5.3 ± 3.1	5.0 ± 3.1	0.09
HOMA-R	1.20 ± 0.81	1.13 ± 0.78	0.12
HOMA-β	96.3 ± 123.8	85.7 ± 93.5	0.11
Decreased insulin secretion^a^: n (%)	20 (4.5)	75 (6.7)	0.11
Systolic blood pressure (mmHg)	123.5 ± 19.6	124.0 ± 18.5	0.64
Diastolic blood pressure (mmHg)	73.4 ± 12.2	73.1 ± 12.1	0.67
Total cholesterol (mg/dl)	204.6 ± 34.1	204.7 ± 33.4	0.96
Triglyceride (mg/dl)	95.4 ± 65.6	96.8 ± 69.2	0.71
HDL cholesterol (mg/dl)	64.6 ± 15.9	65.6 ± 17.4	0.29
Serum albumin (g/dl)	4.5 ± 0.3	4.5 ± 0.3	0.38
Serum uric acid (mg/dl)	5.0 ± 1.3	5.0 ± 1.3	0.48
Serum urea nitrogen (mg/dl)	14.6 ± 4.6	14.3 ± 3.9	0.3
Serum creatinin (mg/dl)	0.71 ± 0.19	0.70 ± 0.15	0.19
AST	22.6 ± 8.4	22.9 ± 8.5	0.49
ALT	21.0 ± 13.3	21.2 ± 14.2	0.74
γGTP	31.0 ± 39.7	33.1 ± 42.7	0.37
Hypertension: n (%)	162 (41.4)	412 (36.8)	0.99
Hyperlipidemia: n (%)	192 (43.6)	498 (44.5)	0.78
Diabetes: n (%)	18 (4.1)	44 (3.9)	0.94
Alcohol consumption: n (%)	180 (40.9)	521 (46.5)	0.046*
Smoking (never/past/current): n	274/87/79	701/220/199	0.99

P < 0.05 is indicated by *. Data presented as mean ± SD or number of subjects (%).

^a^HOMA-β ≤ 30.

**Table 2:** GLP-1R genotype-dependent differnces in nutritional intake.

Nutrient	Men			Women		
	AA–AG	GG	p	AA–AG	GG	p
Energy (kcal/kgBW/day)	32.6 ± 0.82	32.7 ± 0.51	0.92	31.6 ± 0.67	32.4 ± 0.42	0.33
Carbohydrate (g/kgBW/day)	4.45 ± 0.12	4.35 ± 0.08	0.46	4.26 ± 0.09	4.36 ± 0.06	0.37
Fiber (g/kgBW/day)	0.18 ± 0.01	0.17 ± 0.004	0.37	0.20 ± 0.01	0.21 ± 0.004	0.56
Protein (g/kgBW/day)	1.15 ± 0.04	1.14 ± 0.02	0.79	1.24 ± 0.03	1.28 ± 0.02	0.33
Fat (g/kgBW/day)	0.84 ± 0.03	0.83 ± 0.02	0.83	0.96 ± 0.02	0.97 ± 0.02	0.65
Animal (g/kgBW/day)	0.40 ± 0.01	0.40 ± 0.01	0.81	0.43 ± 0.013	0.45 ± 0.01	0.18
Vegetable (g/kgBW/day)	0.44 ± 0.01	0.43 ± 0.01	0.54	0.52 ± 0.013	0.51 ± 0.01	0.55

Data represent mean ± SD.

### Nutrient consumption-dependent association of the GLP1R gene with insulin secretion

We then examined the effect of amounts of nutrients consumed on the association of the GLP1R polymorphism with the index of insulin secretion (HOMA-β) using groups stratified into tertiles based on daily nutrient consumptions (high, middle, and low). As shown in Table 3, HOMA-β of the subjects with the GG genotype tended to be lower compared with subjects with the AA+AG genotypes in most stratified groups, although the differences were not significant.

**Table 3:** Nutrient consumption-dependent association of GLP1R genotype with insulin secretion (HOMA-β).

Nutrient	Men				Women			
	AA+AG	GG	p	Adjusted p	AA+AG	GG	p	Adjusted p
**Energy (kcal/kgBW/day)**								
Low	117.7 ± 13.2	89.0 ± 8.30	0.21	0.08	113.3 ± 13,5	99.8 ± 8.71	0.43	0.45
Middle	82.8 ± 12.7	79.6 ± 7.48	0.90	0.86	103.3 ± 12.8	95.4 ± 8.14	0.51	0.58
High	66.3 ± 7.54	65.2 ± 4.93	0.32	0.36	84.8 ± 7.52	78.1 ± 4.58	0.10	0.34
**Fat (g/kgBW/day)**								
Low	106.0 ± 13.8	82.2 ± 8.1	0.51	0.66	117.4 ± 14.0	100.4 ± 8.75	0.32	0.35
Middle	97.7 ± 11.6	77.1 ± 8.09	0.19	0.07	90.8 ± 11.2	90.9 ± 6.76	0.46	0.70
High	59.4 ± 8.08	71.9 ± 4.73	0.78	0.92	94.5 ± 9.62	81.4 ± 6.32	0.14	0.40
**Animal fat (g/kgBW/day)**								
Low	104.6 ± 12.3	79.4 ± 8.10	0.55	0.77	113.5 ± 12.4	96.6 ± 7.56	0.35	0.32
Middle	76.1 ± 9.19	78.2 ± 5.52	0.84	0.50	94.9 ± 12.4	93.8 ± 8.15	0.36	0.89
High	82.9 ± 12.5	73.7 ± 7.62	0.27	0.10	95.1 ± 10.1	82.6 ± 6.29	0.18	0.47
**Vegetable fat (g/kgBW/day)**								
Low	121.8 ± 14.7	87.3 ± 8.64	0.07	0.06	122.8 ± 16.5	108.0 ± 10.5	0.30	0.37
Middle	86.8 ± 11.4	75.7 ± 7.56	0.97	0.14	96.7 ± 8.95	82.8 ± 5.38	0.13	0.29
High	59.7 ± 6.83	67.9 ± 4.21	0.98	0.60	83.5 ± 7.23	82.2 ± 4.72	0.54	0.82
**Protein (g/kgBW/day)**								
Low	113.1 ± 13.1	83.6 ± 7.81	0.13	0.23	128.5 ± 12.4	99.6 ± 7.62	0.13	0.17
Middle	87.8 ± 12.1	84.4 ± 8.05	0.57	0.36	81.2 ± 11.69	94.7 ± 7.53	0.93	0.66
High	65.8 ± 8.31	63.6 ± 5.01	0.28	0.53	95.3 ± 9.92	78.2 ± 6.23	0.06	0.18
**Carbohydrate (g/kgBW/day)**								
Low	107.0 ± 13.8	91.9 ± 8.12	0.72	0.40	125.9 ± 14.6	98.1 ± 9.81	0.04*	0.08
Middle	89.5 ± 11.5	71.9 ± 7.17	0.55	0.70	86.9 ± 7.15	88.5 ± 4.30	0.80	0.67
High	72.0 ± 8.78	66.6 ± 5.76	0.17	0.09	85.9 ± 11.5	86.8 ± 7.05	0.34	0.54
**Fiber (g/kgBW/day)**								
Low	106.0 ± 7.60	90.8 ± 7.70	0.73	0.71	116.7 ± 13.8	108.0 ± 8.90	0.72	0.86
Middle	91.8 ± 11.6	71.7 ± 7.43	0.35	0.17	106.8 ± 13.1	89.6 ± 7.91	0.13	0.45
High	70.0 ± 9.46	68.2 ± 6.06	0.34	0.45	79.3 ± 6.78	75.4 ± 4.33	0.20	0.23

Data represent mean ± SD or number of subjects (%). Adjusted for age, BMI, and alcohol consumption.

We then evaluated the risk of the genotype for a decreased insulin secretion, which we designated as HOMA-β, ≤ 30 (Table 4). In men, the GG genotype was a significant risk for decreased insulin secretion in most high nutrient consumption groups even after adjustment for multiple factors (age, BMI, alcohol consumption) (e.g. in the high protein consumptions group, OR 15.83, 95% confidence interval 1.58–158.9). Further, significant interaction between the GG genotype and protein consumption was observed as a risk for decreased insulin secretion (p = 0.03). However, such relationships were not observed in any groups stratified in women.

**Table 4:** Risk of GLP-1R genotype (GG) for decreased insulin secretion stratified based on nutrient consumption in (a) men and (b) women.

Nutrient	Univariate			Multiple factors adjusted		
	OR	95% CI	p	OR	95% CI	p
(a) Men						
**Energy (kcal/kgBW/day)**			0.14^#^			0.22^#^
Low	0.77	0.25–2.36	0.65	1.01	0.49–6.26	0.39
Middle	1.84	0.60–5.67	0.29	1.79	0.55–5.80	0.33
High	3.93	1.13–13.6	0.03*	3.95	1.03–15.1	0.04*
**Fat (g/kgBW/day)**			0.24^#^			0.26^#^
Low	0.93	0.36–2.36	0.87	0.95	0.34–2.69	0.93
Middle	3.10	0.67–14.3	0.15	3.34	0.68–16.3	0.14
High	2.93	0.84–10.2	0.09	3.16	0.82–12.3	0.10
**Animal fat (g/kgBW/day)**			0.34^#^			0.24^#^
Low	1.13	0.45–2.88	0.79	1.07	0.39–2.98	0.89
Middle	2.19	0.61–7.79	0.23	2.53	0.65–9.84	0.18
High	3.91	0.88–17.4	0.07	5.82	1.05–32.2	0.04*
**Vegetable fat (g/kgBW/day)**			0.49^#^			0.88^#^
Low	1.43	0.45–4.49	0.54	1.50	0.42–5.33	0.53
Middle	1.36	0.47–3.94	0.57	1.98	0.59–6.63	0.27
High	3.29	0.94–11.4	0.06	2.70	0.69–10.5	0.15
**Protein (g/kgBW/day)**			0.04*^#^			0.03*^#^
Low	1.86	0.52–6.71	0.34	2.79	0.73–10.7	0.13
Middle	0.73	0.27–1.96	0.54	0.78	0.28–2.16	0.63
High	5.94	1.36–25.9	0.02*	15.83	1.58–158.9	0.02*
**Carbohydrate (g/kgBW/day)**			0.39^#^			0.34^#^
Low	1.23	0.38–3.92	0.73	1.63	0.43–6.24	0.48
Middle	1.46	0.52–4.16	0.47	1.23	0.41–3.67	0.72
High	3.57	1.03–12.4	0.046*	4.23	1.10–11.2	0.04*
**Fiber (g/kgBW/day)**			0.07^#^			0.14^#^
Low	0.76	0.22–2.59	0.66	0.99	0.25–3.94	0.99
Middle	1.26	0.43–3.64	0.67	1.29	0.42–3.98	0.65
High	4.95	1.45–17.0	0.01*	4.68	1.27–17.3	0.02*
(b) Women						
**Energy (kcal/kgBW/day)**			0.77^#^			0.76^#^
Low	0.62	0.10–3.75	0.60	0.34	0.04–2.54	0.29
Middle	0.94	0.24–3.71	0.93	1.00	0.25–4.09	1.00
High	1.38	0.38–5.06	0.63	0.99	0.26–3.82	0.99
**Fat (g/kgBW/day)**			0.53^#^			0.50^#^
Low	0.51	0.11–2.34	0.39	0.35	0.07–1.77	0.20
Middle	1.68	0.36–7.92	0.51	1.29	0.26–6.43	0.75
High	1.16	0.3–4.46	0.83	0.82	0.20–3.37	0.78
**Animal fat (g/kgBW/day)**			0.93^#^			0.87^#^
Low	0.74	0.18–3.01	0.67	0.53	0.12–2.38	0.40
Middle	1.31	0.26–6.60	0.74	0.90	0.17–4.95	0.92
High	1.16	0.31–4.39	0.83	0.78	0.19–3.17	0.73
**Vegetable fat (g/kgBW/day)**			0.03*^#^			0.05^#^
Low	0.39	0.10–1.59	0.19	0.27	0.06–1.24	0.09
Middle	NA	NA	NA	NA	NA	NA
High	0.96	0.29–3.19	0.95	0.82	0.24–2.81	0.75
**Protein (g/kgBW/day)**			0.33^#^			0.31^#^
Low	0.37	0.07–1.87	0.23	0.21	0.03–1.29	0.09
Middle	1.90	0.40–8.97	0.42	1.53	0.31–7.59	0.61
High	1.19	0.32–0.22	0.80	0.84	0.21–3.38	0.81
**Carbohydrate (g/kgBW/day)**			0.98^#^			0.98^#^
Low	1.14	0.22–5.95	0.88	0.51	0.08–3.22	0.47
Middle	0.90	0.17–4.73	0.90	0.71	0.13–3.92	0.70
High	1.04	0.32–3.35	0.95	0.83	0.25–2.80	0.77
**Fiber (g/kgBW/day)**			0.16^#^			0.30^#^
Low	0.41	0.06–2.94	0.37	0.24	0.03–2.04	0.19
Middle	0.98	0.25–3.77	0.97	0.67	0.16–2.80	0.59
High	1.52	0.42–5.59	0.53	1.27	0.33–4.86	0.72

*NA* No individual in the AA+AG group in the middle tertile stratified based on vegetable fat consumption was insulin deficient, and, thus, OR could not be calculated.

p < 0.05 is indicated by *. Adjusted for age, BMI, and alcohol consumption.

^#^p-values for interaction between the GLP1R genotype and nutrients in regard with decreased insulin secretion.

## Discussion

In this cross-sectional study of a general Japanese population, we found that the GG genotype of the GLP1R gene polymorphism, rs3765467: A/G or p.Alg131Gln, is a significant risk for decreased insulin secretion in men with high nutrients consumption. Given that the polymorphism was shown to be functional with the allele A associated with an > 100% increase in GLP-1 induced insulin secretion^18,19^, those with the A allele, or the AA+AG genotypes appear to have better insulinotropic action after nutrient consumption, which induces GLP-1 secretion. Therefore, our results are consistent with previous findings, and further indicate that the functional difference between the genotypes may only become evident when nutrient consumption is increased. This observation appears to explain why, clinically, there is a certain subset of the population whose susceptibility to decreased insulin secretion may increase if they consume more nutrients or energy.

We here found that the GG genotype was a significant risk for decreased insulin secretion in the high energy, animal fat, protein, carbohydrate, and fiber consumption groups, but not in the high fat and vegetable fat consumption groups in men. Although all of these nutrients stimulate GLP-1 secretion, the mechanisms involved are different. Carbohydrate or glucose stimulates GLP-1 secretion from L-cells through mechanisms similar to the stimulation of insulin secretion in the islets, or through glucose–mediated membrane depolarization, while protein and lipid bind specific cell surface receptor of L cells to stimulate GLP-1 secretion^20,21^. Further, although ingestion of carbohydrates or proteins elicits a rapid increase in circulating GLP-1 with a peak 30–60 min following nutrient intake, ingestion of fat elicits a more prolonged (> 120 min) increase^33,34^. Therefore, differences in time profile after nutrient intake and circulating levels of GLP-1 secreted depending on each nutrient consumed may be responsible for the observed differences in nutrient consumption-dependent association. However, the difference might merely come from low statistical power of the analysis, as the subjects were stratified based on nutrient intake, thus, the number of subjects in each group became small, and the risk of the GG genotype for decreased insulin secretion in the high fat and vegetable fat consumption groups were, though not significant, positive [OR 3.16 (0.82–12.3) and 2.70 (0.69–10.5), respectively] with marginal significance. This issue needs to be evaluated in the future.

The nutrient consumption-depending association between the genotype and decreased insulin secretion were observed only in men. Sex hormones are shown to induce GLP-1 secretion and also to modulate effects of GLP-1 on glucose homeostasis and food intake through their interaction to GLP1R^35^. Further, less sensitivity to liraglutide, a GLP-1 derivative, in men has been suggested from clinical trials^36^. Together, these facts may explain the observed gender-specific difference in the nutrient consumption-depending association between the genotype and decreased insulin secretion**,** though the underlying explanation in details is awaited. Alternatively, the fact that the number of subjects with a decreased insulin secretion was much lower in women than in men [n (%): 29 (3.0) vs. 66 (11.2)], may be the cause of the observation, as this may have resulted in low statistical power. Indeed, HOMA-β of subjects with the GG genotype tended to be lower compared with subjects with the AA+AG genotypes in both women and men. Therefore, a similar nutrient-dependent association may also exist in women.

As described previously, DM is considered multifactorial, and, thus, a lifestyle-related disease, as many genetic and environmental factors are involved together in its pathophysiology^1,2^. Namely, effects of genetic factor may become evident only when their corresponding environmental factors are accompanied. Conversely, without considering such environmental factors, true effects of genetic factors may not be evaluated either precisely or effectively. Mice without a gene (knock out mouse) involved in glucose metabolism such as adiponectin showed impaired glucose tolerance only when they were fed with high fat and high sucrose chow, but not with normal chow^37^. Further, in a human study, gene polymorphisms in TCF7L2, which is a well-annotated gene associated with DM found in various studies including GWAS^38,39^, was shown to be associated with an increased risk of diabetes among persons with impaired glucose tolerance in groups without lifestyle intervention but not with lifestyle intervention^40^. Therefore, finding such environmental factors corresponding to each genetic factor appears to be important, as such information can be implicated to develop possible intervention means in the general clinical setting. The results found here seem to suggest that there is a certain subset of the population whose susceptibility to decreased insulin secretion may increase if they consume more nutrients such as, at least, protein. Glucose-dependent insulinotropic polypeptide (GIP) is another major incretin hormone beside GLP-1 and augments insulin secretion after a food intake, and its release is influenced by various nutrients consumed^41–43^. Therefore, nutrient consumption-dependent association of GIP or its receptor (GIPR) with DM or decreased insulin secretion is also interesting to be evaluated. In this regard, to date, two studies examined nutrient consumption-dependent association of a GIPR gene polymorphism (rs10423928) with DM^44–46^. A report showed that subjects with the AA genotypes of the polymorphism (the A allele is associated with lower insulin secretion after an oral glucose tolerance test^47^) consuming high-fat low-carbohydrate diets had reduced risk of DM^44^, while another showed no such association^41^. The results of the former study appear to be different from our results, where high nutrients consumption including fat is also a risk for decreased insulin secretion in the genotype depending manner. Different from GLP-1, GIP has a role in fat accumulation in adipocytes^44,48^, and, thus, amount of fat consumed may influence directly or indirectly (may through obesity) to glucose tolerance differently, leading to the different nutrient consumption-depending association observed here.

We here examined nutrient consumption-dependent association without considering eating habits, which may affect glucose tolerance more than amount of each nutrient per se^49^. Further, not only so-called bad eating habits such as skipping breakfast, eating late, and consuming greater amount of food, but also sequence to consume meal has influence on glucose tolerance^49^. Namely, preloading of a protein-rich diet before carbohydrate intake increases secretion of GLP-1 and GIP, and enhance early-phase insulin secretion^50,51^. Therefore, studies in consideration of such eating habits are awaited to precisely evaluate nutrient consumption-dependent association of the GLP1R gene with decreased insulin secretion.

We did not observe an association between the genotype and DM per se (Table 1). To precisely evaluate HOMA indices, we excluded subjects on medication for DM and with FBG levels below 63 mg/dl or over 140 mg/dl. Therfore, the number of subjects with DM in the study population was small [n = 62 (4.0%)]. This may explain the nonsiginificant association between the genotype and dibetes, as the small number of subjects and the exclusion may have led to low statistical power and selection bias. However, even when include those excluded from the study [the number of subjects with DM increased to 160 (9.5%)], no siginificant association between the genoype and DM was observed [number of diabetic subject with the AA+AG genoypes vs. GG genoype: 46 (9.7%) vs. 114 (9.5%), p = 0.90]. Analyses with subjects stratified based on nutrient intake did not reveal a significant association between the genotypes either. Therefore, although such an association has been reproted in several case–control studies with relatively large sample-sizes^13–18^, the effect of the genotype on development of DM may not be substantial as it can be evaluated in studies of the general population with a relatively small sample size.

The proportion of subjects who were currently alcohol drinkers was higher in those with the GG genotype than in those with the AA+AG genotypes. GLP-1 is also synthesized in neurons of the hindbrain, acts as a neuropeptide, and, regulates food intake and appetite^52–55^. Accordingly, its abilities to control alcohol consumption have also been reported in both animals and humans^52,56–60^. GLP1R agonists decreased the motivation to consume alcohol in rodents and monkeys^52,56–60^, and an association between polymorphisms of the GLP1R gene and alcohol dependency was reproted in a case–control study of patients with alcohol use disoder (AUD)^60^. Therefore, the association between the GG genotype and habitual alcohol consumption appears to be consistent with these prior studies, although the human study evaluated patients of Afro Americans and Caucasion descent with AUD, while we evaluated a general Japanes population. As reproted, the GG genotype appear to has lower ability to transduct signals of GLP-1 compared to the AA+AG genotypes^18,19^, the effects of GLP-1 to decrease the motivation to consume alcoohl might be decreases in subjects with the GG genotype. Together, our result seems to indicate that the genotype GG is a risk for habitual alcohol consumption in both the Japanese and general populations.

The present study had both strengths and limitations. Strengths were as follows. The statistical adjustments were made for multiple factors that could have confounded the results, and the general population-based sample was analyzed. In addition, subjects on medication for DM were excluded, as these drugs affect glycemic parameters including HOMA indices. Subjects with FBG levels below 63 mg/dl or over 140 mg/dl were excluded to precisely evaluate HOMA indices. Thus, the results obtained appear to precisely reflect the relationship between the genotypes and HOMA indices. Limitations were as follows. The participants were selected from a health promotion study and not from a population undergoing ordinary health check-ups, and thus the participants may have been more invested in their health than the general population. Therefore, subjects may not accurately represent the general population. Further, we used HOMA-β to evaluate insulin secretion ability. However, HOMA-β represents β-cell function in the fasting state, not in response to nutritional stimulation. Further, major factors related to nutrient consumption, gender and age were adjusted differently: i.e. gender was used for stratification, and age was used for statistical adjustment as a continuous variable. Namely, stratification based on age might bring different results. However, as univariate regression analyses showed that amount of nutrient consumed appeared to increase along with age without any obvious reflection point (energy: β = 0.187, p < 0.0001 and β = 0.178, p < 0.0001, for men and women, respectively), the possibility does not seem to be substantial. Furthermore, we here examined a nutrient consumption-dependent association of a GLP1R gene polymorphism with insulin secretion in regard with macronutrients but not with micronutrient. BDHQ give values for huge number of micronutrient consumed. Therefore, we could examine the association also in regard with micronutrient. However, as an initial step of this association study, we concentrated to examine the association in regard with macronutrients only to simplify the analyses or to avoid an issue of multiple testing. In addition, several information, which appear to be useful to explore the association more in depth, such as menopausal status and plasma GLP-1 levels were not evaluated. Since measuring plasma GLP-1 levels requires quick handling of blood samples using tubes with DPP-4 inhibitors and protease inhibitors, and, thus, could not be appreciable for ordinary healthcare examinations, we did not measure plasma GLP-1 levels, and, thus, could not evaluate differences in nutrient consumption-dependent association with decreased insulin secretion between the GLP1R genotype and plasma GLP-1 levels, which may lead to more detailed explanation of the pathophysiology of such association. Finally, as our study was cross-sectional and not a cohort study, we could not assess whether the GG genotype is a risk for future decrease in insulin secretion or eventually incidence of DM.

In conclusion, a GLP1R gene polymorphism was associated with decreased insulin secretion, but only in men with high energy, animal fat, protein, carbohydrate and fiber consumption in Japanese, suggesting a nutrient consumption-dependent association between the gene and decreased insulin secretion.

## Data Availability

All data generated or analyzed during this study are included in this published article.
